# Equivalent Electromagnetic Constants for Microwave Application to Composite Materials for the Multi-Scale Problem

**DOI:** 10.3390/ma6115367

**Published:** 2013-11-21

**Authors:** Keisuke Fujisaki, Tomoyuki Ikeda

**Affiliations:** 1 Toyota Technological Institute, 2-12-1 Hisakata, Tenpaku-ku, Nagoya-city 468-8511, Japan; 2 Kojima Press Industry Co. Ltd., 15 Hirokuden, Ukigai-cho, Miyoshi-city 470-0207, Japan; E-Mail: t-ikeda@kojima-tns.co.jp

**Keywords:** multi-scale, equivalent material constant, eddy current, electrical conductivity

## Abstract

To connect different scale models in the multi-scale problem of microwave use, equivalent material constants were researched numerically by a three-dimensional electromagnetic field, taking into account eddy current and displacement current. A volume averaged method and a standing wave method were used to introduce the equivalent material constants; water particles and aluminum particles are used as composite materials. Consumed electrical power is used for the evaluation. Water particles have the same equivalent material constants for both methods; the same electrical power is obtained for both the precise model (micro-model) and the homogeneous model (macro-model). However, aluminum particles have dissimilar equivalent material constants for both methods; different electric power is obtained for both models. The varying electromagnetic phenomena are derived from the expression of eddy current. For small electrical conductivity such as water, the macro-current which flows in the macro-model and the micro-current which flows in the micro-model express the same electromagnetic phenomena. However, for large electrical conductivity such as aluminum, the macro-current and micro-current express different electromagnetic phenomena. The eddy current which is observed in the micro-model is not expressed by the macro-model. Therefore, the equivalent material constant derived from the volume averaged method and the standing wave method is applicable to water with a small electrical conductivity, although not applicable to aluminum with a large electrical conductivity.

## 1. Introduction

Microwave heating is characterized by the ability to heat rapidly, effectively and selectively. It causes anomalous phenomena, which are raising of the boiling temperature [1], changing the conditions of chemical reaction [2], promoting the nitriding reaction [3] and the reduction reaction [4], azotizing titanium under atmospheric pressure [5], *etc*. It was reported as an incredible and surprising phenomenon that powdered metal could be heated by microwave [6], because it was generally considered that metal reflects the electromagnetic field and is not possible to be heated. This phenomenon introduced the possibility of microwave application to the metal industry and promoted further research for metallurgists [7,8].

The metal industry is one of the major energy consumers and is expected to reduce energy as well as CO_2_ discharge [9]. A deoxidization phenomenon of iron oxide, which is an ingredient of steel products, was discovered when microwave was applied to iron oxide [10], and subsequently a one-ton plant per day was built and succeeded as a test plant [11].

The electromagnetic phenomenon of why microwave is possible to be used to heat the metal and to promote the deoxidization phenomenon is said to be one of the most important scientific problems to be clarified, because microwave application is mainly researched on experimental data, whereas electromagnetic phenomenon elucidation is not enough. Historical facts say that industrial problems often contribute significantly to physical scientific progress. The temperature measurement of molten steel was discussed in the steel making plant and quantum mechanics was created about 100 years ago.

**Figure 1:**
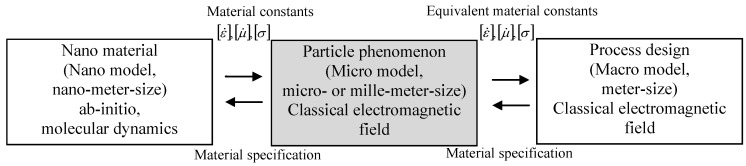
Multi-scale phenomenon of microwave application.

Consequently the numerical calculation of electromagnetic field analysis taking into account eddy currents [12,13,14] and molecular dynamics analysis [15,16] were researched. They were considered to contribute to the fundamental analysis of microwave application. However, since the microwave application phenomenon is observed not only in nano-scale but also in macro-scale, the point of view of multi-scale is considered to be important. It makes it possible to construct different scale models in order to clarify the electromagnetic field phenomenon in detail and to design the materials or particles as well as the process [17]. The details are shown in Figure 1.

Fundamental electromagnetic phenomena and material constants are derived from nano-scale research. To realize and to design the manufacturing process macro-model is useful. However, because the nano-model is too small to be treated in macro-model scale, in designing and calculating numerically, the micro-model is expected to have an important role in connecting the nano-model and macro-model by using the equivalent material constants. Since the material is usually shaped as a particle type its shape often has an influence on the electromagnetic field phenomenon [18]; the micro-model to express the particle shape is also important. To represent the electromagnetic phenomenon in different scale models and to connect up the nano-scale, micro-scale and macro-scale, the physical equivalent electromagnetic material constants in the micro-model are an indispensable problem which needs to be considered.

The micro-model uses two methods to obtain the material constants. One is a volume averaged method and the other is a standing wave method. The volume averaged method is used in physical theory [19,20,21,22] or in measured representative data of magnetization material [23,24]. The standing wave method is used in the measured electromagnetic material constants in the electromagnetic wave, where a network analyzer is used [25,26]. The material constants derived from the two methods should be equal.

Here, a three-dimensional numerical electromagnetic field analysis is researched theoretically for comparison, where two kinds of materials such as water and aluminum are considered. The details are shown in Figure 1.

## 2. Calculation Methods

### 2.1. Finite Element Method (FEM) Analysis

In the micro model, the particle size of the material is considered to be small enough compared with the wave length of the electromagnetic field. A micro-sized model of the electromagnetic field is used here [14]. It is assumed that a fundamental electromagnetic particle structure is considered and the same particle structure continues repeatedly in three dimensional space infinitely. The finite element method (FEM method) with the A-φ method and *j*ω method is used here, because the finite-difference time-domain method (FDTD method) needs a lot of calculation time-divisions to pass though the particle materials and takes a great deal of CPU-time, of the order of a century or more. Eddy current, and displacement current are taken into account and the basic equations can be expressed as follows:

(1) $$rot\frac{1}{\mu }rotA=-(\sigma +J{\omega \varepsilon }_{0}\varepsilon_{r}^{\prime })grad\phi +j\omega A)$$

Here, μ, $\varepsilon_{0}\varepsilon_{r}^{\prime }$ , σ are magnetic permeability, real part of dielectric constant and electrical conductivity respectively $j=\sqrt{-1}$ . ***A*** and ϕ is the magnetic vector and the magnetic potential can be defined as follows:

(2) rotA=B, E=−gradφ−jωA

Here, *B* and *E* are magnetic flux density vector and electrical field vector respectively. Bold character means a vector, and ω means the angular frequency of the electromagnetic field. A value of 2.45 GHz is considered as an electromagnetic field frequency.

### 2.2. Boundary Condition

**Figure 2:**
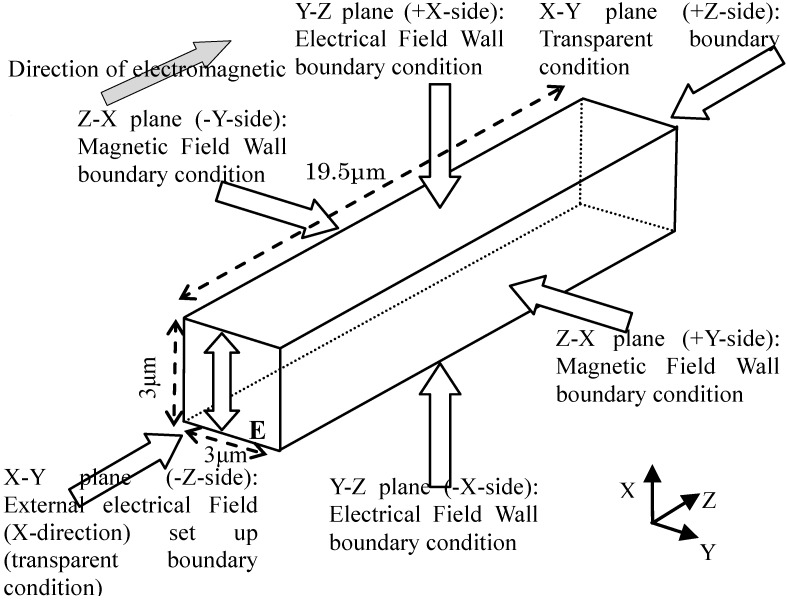
Boundary conditions of a micro-sized numerical model.

Since the fundamental structure repeats, the boundary condition of the electromagnetic field numerical calculation is shown in Figure 2, where the electromagnetic wave is assumed to be travelling in the repeated direction. There are six boundaries in the model.

The *Z*-*X* plane in the –*Y*-side is set up to be a transparent boundary condition, where an external electrical field is set up in the *X*-direction and the electromagnetic field is travelling in the +*Z* direction. The *Z*-*X* plane in the +*Y*-side is set up to be a transparent boundary condition, where the electromagnetic field passes though. Both *Y*-*X* planes are set up to be an electrical field wall boundary condition. Both *Z*-*X* planes are set up to be a magnetic field wall boundary condition.

The external electrical field is considered to be set up as 1 V/m, and the external magnetic field is derived from the next equation. The boundary conditions in the *Z*-*X* plane in the –*Y*-side are shown in Table 1.

(3) $$E_{x}=\sqrt{\frac{\mu }{\varepsilon }}H_{y}$$

**Table 1:** Boundary conditions of a micro-sized model.

Input data	Symbol	Unit	Real part	Imaginary part
Electrical field (*X* component)	*E_x_*	V/m	1.0	0.0
Electrical field (*Y* component)	*E_y_*	V/m	0.0	0.0
Electrical field (*Z* component)	*E_z_*	V/m	0.0	0.0
Magnetic field (*X* component)	*H_x_*	A/m	0.0	0.0
Magnetic field (*Y* component)	*H_y_*	A/m	2.65 × 10^−3^	0.0
Magnetic field (*Z* component)	*H_z_*	A/m	0.0	0.0

### 2.3. Particle Structure

**Figure 3:**
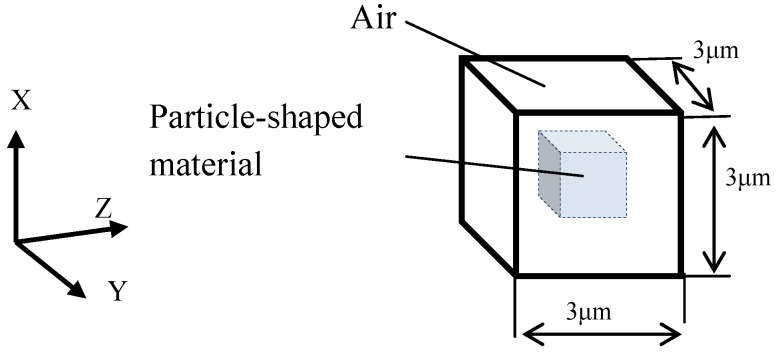
The fundamental structure used here continues repeatedly in three-dimensional space.

The fundamental structure with a particle is considered as in Figure 3, which is 3 μm cubic and has particle material. Outside of the particle is air. Material volume rates of 20% and 80% are considered for comparison. The material of the particles is water and aluminum. Electromagnetic material constants are shown in Table 2. The relative dielectric constant and relative magnetic permeability is defined as in the following equation.

(4) $$\mu =\mu_{0}\mu_{r}^{\prime }+j\mu_{r}^{\prime \prime },\varepsilon =\varepsilon_{0}(\varepsilon_{r}^{\prime }+j\varepsilon_{r}^{\prime \prime })$$

Here, μ_0_ and ε_0_ are the permeability and dielectric constants of vacuum respectively. The electrical conductivity and imaginary part of the relative dielectric constant are related as in the next equation, where the water’s electrical conductivity of the DC (direct current) component is not considered.

(5) $$\sigma =-2\text{π}f\varepsilon_{0}\varepsilon_{r}^{\prime \prime }$$

The electromagnetic characteristics of the particle are isotropic and homogeneous. The skin depth is defined as in the next equation.

(6) $$\delta =\sqrt{\frac{1}{\text{π}f{\sigma \mu }_{\text{0}}\mu_{r}^{\prime }}}$$

**Table 2:** Material constants in the micro-sized model.

Material constants		Unit	Symbol	Air	Water	Aluminum
Relative magnetic permeability	Real	–	$\mu_{r}^{\prime }$	1	1	1
	Imaginary	–	$\mu_{r}^{\prime \prime }$	0	0	0
Relative dielectric constant	Real	–	$\varepsilon_{r}^{\prime }$	1	76.7	1
	Imaginary	–	$\varepsilon_{r}^{\prime \prime }$	0	−12.04	−2.601 × 10^8^
Electric conductivity		S/m	σ	0	1.608	3.700 × 10^7^
Skin depth (2.45 GHz)		mm	δ	0	8.02	1.67 × 10^−3^

The skin depth of water is much larger than the water particle size. Therefore the eddy current can not flow within the particle so as to make a circle around the Y-direction. However, the skin depth of aluminum is almost the same as the aluminum particle size. Therefore in this case it is possible for the eddy current to flow in the particle so as to make a circle around the Y-direction [14].

**Figure 4:**
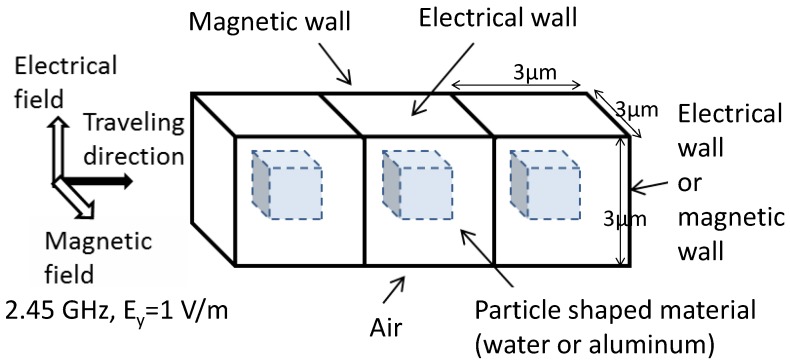
Micro-scale model in electromagnetic field.

In the electromagnetic numerical calculation, the 3-particles shown in Figure 4 are used here in order to avoid the end effect of the electromagnetic field as input and output. Three-dimensional space in the FEM analysis is divided into about 1,000,000 meshes. The electromagnetic FEM calculation makes electromagnetic field vectors in each mesh.

### 2.4. Post Processing

The electromagnetic vectors are used for the post processing in order to obtain the equivalent material constants.

#### 2.4.1. Volume Averaged Method

The volume averaged method is described in electromagnetic theory in [15] by Landau and Lifshitz. Here, it is applied to the equivalent material constants of the electromagnetic field.

After the electromagnetic numerical calculation, the electromagnetic physical values are introduced as, *E_xi_*, *D_xi_*, *B_yi_*, *H_yi_*, which are the electrical field, dielectricflux density, magnetic flux density and magnetic field respectively. Suffix of “*x*” and “*y*” means *X*-component and *Y*-component respectively and suffix “*i*” means mesh number in the divided FEM mesh. *V_i_* means a volume of the *i*-th mesh. From which the volume averaged electromagnetic physical values are introduced as in the next equation.

(7) $$E_{xave}=\frac{\sum E_{xi}V_{i}}{\sum V_{i}},\;D_{xave}=\frac{\sum D_{xi}V_{i}}{\sum V_{i}},\;B_{yave}=\frac{\sum B_{yi}V_{i}}{\sum V_{i}},\;H_{yave}=\frac{\sum H_{yi}V_{i}}{\sum V_{i}}$$

Here, since the *X*-component of the electrical field and the *Y*-component of the magnetic field are given as an external electromagnetic field, only their components are considered. The summation is calculated only for the center particle of the 3-particles as shown in Figure 4.

From the averaged electromagnetic physical values, the equivalent electromagnetic material constants can be introduced by the next equation.

(8) $$\varepsilon_{r}=\frac{1}{\varepsilon_{0}}\frac{D_{xave}}{E_{xave}},\;\mu_{r}=\frac{1}{\mu_{0}}\frac{B_{yave}}{H_{yave}}$$

The volume averaged method was also applied to the macro model of magnetic shielding and has quite good agreement with data from measurement [20,21]. Energy conservation between the micro- and macro-model is discussed in [27,28,29]. Magnetic domain has an important role in the magnetic body and the magnetic field and magnetic flux density were measured by the volume averaged method [23,24,30].

#### 2.4.2. Standing Wave Method

In the standing wave method, the same data processing used in the network analyzer is calculated here numerically. In the network analyzer, input energy, reflect energy and transparent energy are measured, and then the equivalent electromagnetic material constants are introduced from transmission line theory as follows [25,31].

(9) $$\varepsilon_{r}=\frac{c}{2\text{π}fl}\frac{Z_{0}}{Z_{op}}\tan^{-1}\sqrt{-\frac{Z_{sh}}{Z_{op}}},\;\mu_{r2}=\frac{Z_{sh}Z_{op}}{Z_{0}^{2}}\varepsilon_{r}$$

Here, Z0=μ0ε0 is the characteristic impedance in vacuum, *Z_sh_* is the input impedance when the terminal impedance is short, *Z_op_* is the input impedance when the terminal impedance is open. c=1μ0ε0 is the plane wave velocity in vacuum.

In the FEM analysis of electromagnetic field as in Figure 4, *Z_sh_* and *Z_op_* correspond to the input impedance of the electric wall boundary condition and the magnetic wall boundary condition at the *X-Y* plane (+*Z*-side) in Figure 2, respectively.

### 2.5. Evaluation

The introduced equivalent electromagnetic material constants should be evaluated in comparison with the precise model. The precise model uses a model, Figure 4, where the particle shape and its material constants are taken into account. The equivalent material constants are considered to be applied to the homogeneous model, where the outer shape is the same as in Figure 4. The material constants within the homogeneous model are uniform, and the introduced equivalent electromagnetic material constants are applied. As for the multi-scale problem, as in Figure 1, the precise model corresponds to the micro-model, and the homogeneous model corresponds to the macro model.

The precise model and the homogenous model should have the same electromagnetic characteristics, although the electromagnetic distribution within the model is quite different. Therefore, electromagnetic heating within the model is used for comparison data where the input electromagnetic field condition is the same. The electromagnetic heating value in the particle can be introduced by the next equation.

(10) $$P_{\text{heat}}=\sigma {\left|E\right|}^{2}$$

## 3. Calculation Results

### 3.1. Water and Air

**Figure 5:**
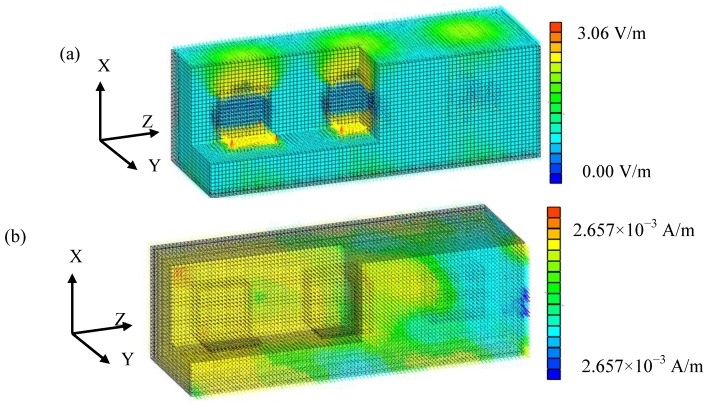
Electromagnetic field calculation result (water, volume rate: 10%). (**a**) Electrical field, real part. (**b**) Magnetic field, real part.

In the case of particle-shaped water, the electromagnetic field distribution is shown in Figure 5. The electrical field in the real part mainly has an *X*-component y and has a larger value than the input external electric field in water, because it is ferroelectric. The magnetic field in the real part has mainly a *Y*-component and uniform distribution. Its value is the same as the external magnetic field, because water is not ferromagnetic and not electrically conductive.

The equivalent material constants derived from Equations (8) and (9) are shown in Table 3 and Table 4, where the water volume rate is 10% and 80%, respectively.

**Table 3:** Equivalent physical properties of particle-shaped water (water, volume rate: 10%).

Equivalent physical properties	Volume Averaged method	Standing Wave method	Deviation (%)
$\varepsilon_{r}^{\prime }$	1.395	1.401	0.4
$\varepsilon_{r}^{\prime \prime }$	−0.004	−0.004	0.0
$\mu_{r}^{\prime }$	0.999	0.975	−2.4
$\mu_{r}^{\prime \prime }$	0.000	0.000	0.0

**Table 4:** Equivalent physical properties of particle-shaped water (water, volume rate: 80%).

Equivalent physical properties	Volume Averaged method	Standing Wave method	Deviation (%)
$\varepsilon_{r}^{\prime }$	12.074	12.033	−0.3
$\varepsilon_{r}^{\prime \prime }$	−0.312	−0.310	−0.6
$\mu_{r}^{\prime }$	0.999	1.000	0.1
$\mu_{r}^{\prime \prime }$	0.000	0.000	0.0

The relative dielectric constants of the real part and the imaginary part have the same value for the volume averaged method and the standing wave method. This tendency is observed for both 10% and 80% volume rate. However, the numerically calculated relative dielectric constants in the real part and imaginary part themselves are not equal to the values from multiplying the material constants by the volume rate. The former is smaller than the latter. The generation of a depolarization field within the micro-structured water causes the decrease of the electrical field [18,32].

The relative magnetic permeabilities of the real part and the imaginary part have also the same value as air for the volume averaged method and the standing wave method. Water is not ferromagnetic and not electrically conductive. Since the equivalent physical material constants of the volume averaged method and the standing wave method are almost the same, the values of the volume averaged method are used here for the evaluation with the precise model.

The equivalent physical material constants of Table 3 and Table 4 are applied to the homogeneous model, and then the electrical heating value is calculated from Equation (10). It is the consumed electrical power, and is shown in Table 5, where the water volume rate is 10% and 80%, respectively. The consumed electric powers introduced by the equivalent material constants have almost the same values as the precise model for 10% and 80% water volume rate.

Therefore, it can be concluded that both the volume averaged method and the standing wave method are effective methods for the introduction of the equivalent material constants.

**Table 5:** Consumed electrical power in the precise model and homogeneous model.

Calculation data	Precise model	Homogeneous model	Deviation (%)	Water volume rate
Consumed electrical power (W)	1.93 × 10^−20^	1.94 × 10^−20^	0.5	10%
	1.58 × 10^−18^	1.68 × 10^−18^	5.9	80%

### 3.2. Aluminum and Air

**Figure 6:**
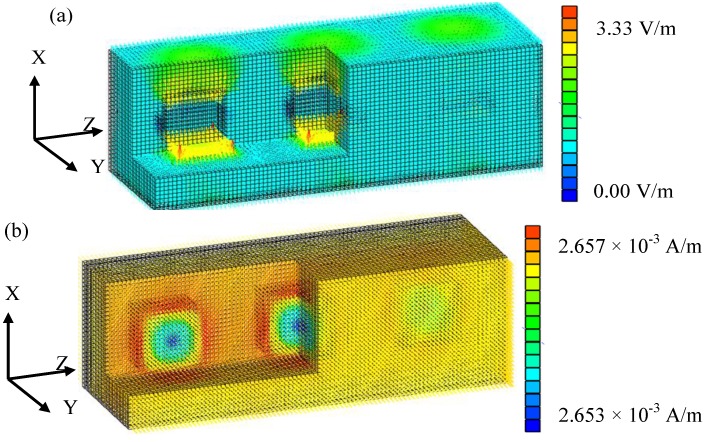
Electromagnetic field calculation result (aluminum, volume rate: 10%). (**a**) Electrical field, real part. (**b**) Magnetic field, real part.

In the case of particle-shaped aluminum, the electromagnetic field distribution is shown in Figure 6. The electrical field in the real part has mainly an *X*-component and has a larger value than the input external electric field, because the electrical field flows in a detour around the aluminum. The magnetic field in the real part mainly has a *Y*-component without uniform distribution. The center part of the aluminum has a small magnetic field, because of the eddy current induced in the aluminum. Since aluminum is electrically conductive, the time variation of the magnetic field (magnetic flux density) causes the eddy current around the Y-direction [14]. The eddy current introduces a magnetic field so as to deny the external magnetic field. Therefore, the magnetic field is distributed.

The equivalent material constants derived from Equations (8) and (9) are shown in Table 6 and Table 7, where the water volume rates are 10% and 80%, respectively.

The relative dielectric constants of the real part and the imaginary part are different between the volume averaged method and the standing wave method. This tendency is observed for both 10% and 80% volume rates. The numerically calculated relative dielectric constants of the imaginary part are themselves quite different from the material constants of Table 2. Here the relative dielectric constant of the imaginary part is related to the electric conductivity as in Equation (5). Since the aluminum particle electrically insulates because of air around the particle, different particles are not electrically conductive. So it is reasonable to say that the electrical conductivity of the homogeneous model, which is the equivalent to the relative dielectric constants of the imaginary part, is much smaller (insulating).

The relative magnetic permeabilities of the real part have also have the same value as air for the volume averaged method and the standing wave method. Aluminum is not ferromagnetic. The relative magnetic permeability of the imaginary part is observed in the standing wave method. The eddy current appears to cause it.

The equivalent physical material constants from Table 6 and Table 7 are now applied to the homogeneous model, and then the electrical heating value is calculated from Equation (10). The consumed electric powers are shown in Table 8, where the aluminum volume rates are 10% and 80%, respectively. All the consumed electric powers are different between the precise model and the homogeneous model which uses the equivalent physical material constants of the volume averaged method and the standing wave method for 10% and 80% aluminum volume rates.

**Table 6:** Equivalent physical properties of particle-shaped aluminum (volume rate: 10%).

Equivalent physical properties	Volume Averaged method	Standing Wave method	Deviation (%)
$\varepsilon_{r}^{\prime }$	1.417	9.831	593
$\varepsilon_{r}^{\prime \prime }$	−0.001	−0.055	(5400)
$\mu_{r}^{\prime }$	0.999	1.000	0.1
$\mu_{r}^{\prime \prime }$	0.000	−0.005	–

**Table 7:** Equivalent physical properties of particle-shaped aluminum (volume rate: 80%).

Equivalent physical properties	Volume Averaged method	Standing Wave method	Deviation (%)
$\varepsilon_{r}^{\prime }$	14.464	10.878	−24
$\varepsilon_{r}^{\prime \prime }$	0.002	0.077	(3750)
$\mu_{r}^{\prime }$	0.999	0.965	−3.4
$\mu_{r}^{\prime \prime }$	0.000	−0.145	–

**Table 8:** Consumed electrical power in the precise model and homogeneous models.

Calculation data	Precise model	Homogeneous model (Volume Averaged method)	Homogeneous model (Standing Wave method)	Aluminum, volume rate
Consumed electrical power (W)	2.64 × 10^−20^	0.55 × 10^−20^	32.8 × 10^−20^	10%
	80 × 10^−20^	1 × 10^−20^	122 × 10^−20^	80%

The differences in the equivalent material constants, as well as the differences in consumed electric power, are considered as follows.

1. Eddy current effect; the eddy current flows inside the aluminum particle because of the external magnetic field and then it produces new components of the magnetic field and electrical field. The new components seem to make the magnetic field and electrical field different from the external electromagnetic field as well as having an influence on the equivalent material constants.
2. Much smaller electrical conductivity; since each particle is insulated, the eddy current of a set of particles becomes much smaller. This means that the equivalent material constants of the electrical conductivity become much smaller, and then the effect of the eddy current which flows inside the particle can be ignored. Therefore a different electromagnetic phenomenon is observed between the precise model and the homogeneous model.

The explanation “2” above shows the importance of the equivalent electrical conductivity. Since the eddy current flow appears within the aluminum particle locally, the homogenous model does not seem to be able to express it. The eddy current is reported as having an important role in order that the electromagnetic field inserts particle-shaped metal [12,14]. Therefore, it may be more useful for the homogeneous model to express the electromagnetic phenomena such that the local eddy current exists within the metal particle.

It can be concluded that special attention for the electromagnetic phenomenon is required in order to consider the equivalent material constants for the composite material with metal (electrical conductive material).

### 3.3. Discussion

The electromagnetic homogeneous model which uses the equivalent material constants is useful for ferroelectric bodies such as water. The equivalent material constants derived from the volume averaged method and the standing wave method have almost the same values, and the consumed electric power by the homogenous model coincides with that of the precise model.

However, the electromagnetic homogeneous model which uses the equivalent material constants is not useful for particle-shaped electrical conductivity. The equivalent material constants derived from the volume averaged method and the standing wave method are quite different, and the consumed electric power by the homogenous model and the precise model are also quite different.

The difference is considered to be derived from the eddy current and the electric charge distribution. According to reference [14], eddy current distribution depends on the electrical conductivity, particle shape and frequency.

**Figure 7:**
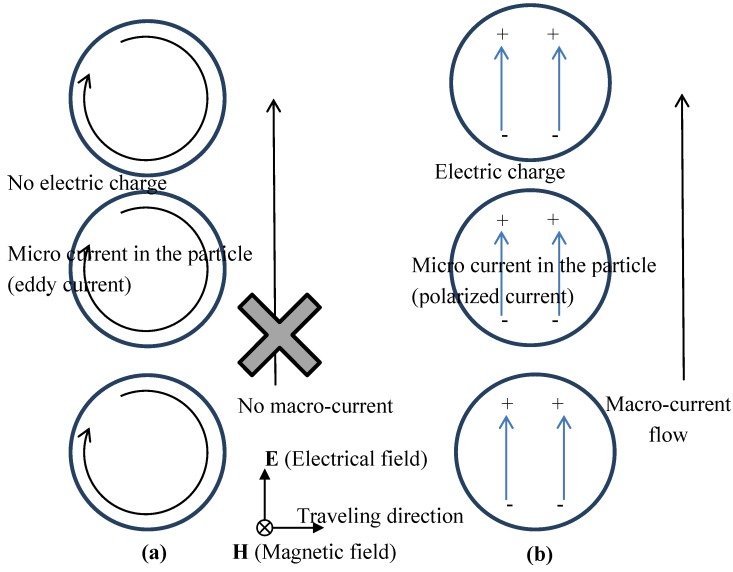
Current flow and electric charge distribution. (**a**) Large electrical conductivity such as metal. (**b**) Low electrical conductivity such as water.

When the particle-shaped material has a large electrical conductivity such as aluminum, micro-current flows in the particle-shaped material so as to make a circle around the H-field direction as shown in Figure 7a. This micro-current is usually called eddy current. When the particle-shaped material has a small electrical conductivity such as water, the micro-current flows in one direction (the electrical field direction) in the particle as shown in Figure 7b. This micro-current is usually called a polarized current.

With large electrical conductivity, the continuity of micro-current is guaranteed. So the electric charge does not appear within the particle. Therefore, a macro-current, which is defined here as a current that flows between adjacent particles, is not observed, as shown in Figure 7a. Only the macro-current is effective in the homogeneous model, because it is impossible to express micro-current in the homogeneous model. Therefore, it can be said that the homogeneous model has difficulty in expressing eddy current except in special modeling.

With small electrical conductivity, the continuity of micro-current is not guaranteed. So the electric charge appears within the particle. Therefore, a macro-current is observed by way of electric charge as shown in Figure 7b. The macro-current is said to be almost the same as the micro-current. Hence, it can be said that the homogeneous model seems to express the micro-current well.

As for the multi-scale problem which connects macro-scale and micro-scale, the eddy current which flows in the micro-scale is more difficult to express in the macro-scale. So the equivalent electrical conductivity, which also corresponds to the relative dielectric constant of the imaginary part as Equation (5), is more difficult to introduce in the macro-scale, when the eddy current is generated in the micro-scale. There seems to be a limitation in the electromagnetic theory to express eddy current by a material constant as an electrical conductivity.

## 4. Conclusions

To connect the different scale models in the multi-scale problem of the microwave, equivalent material constants have been researched numerically by a three-dimensional electromagnetic field taking into account both eddy current and displacement current. The volume averaged method and the standing wave method were used to introduce the equivalent material constants, and the water particle together with the aluminum particle were used as composite materials for comparison. Both methods and the different scale models were evaluated by the consumed electrical power.

The water particle has the same equivalent material constants with both methods, and the same electrical power is obtained for both the precise model (micro-model) and the homogeneous model (macro-model). However, the aluminum particle has different equivalent material constants for both methods, and different electric power is obtained for both models. 

The different electromagnetic phenomena are derived from the expression of eddy current. For small electrical conductivity such as water, the macro-current which flows in the macro-model and the micro-current which flows in the micro-model express the same electromagnetic phenomena. However, for large electrical conductivity such as aluminum, the macro-current and the micro-current express different electromagnetic phenomena. The eddy current which is observed in the micro-model is not expressed by the macro-model.

Therefore, the equivalent material constant derived from the volume averaged method and the standing wave method is applicable to water with a small electrical conductivity, although not applicable to aluminum with a large electrical conductivity.
